# Nanoformulated Antiretroviral Therapy Attenuates Brain Metabolic Oxidative Stress

**DOI:** 10.1007/s12035-018-1273-8

**Published:** 2018-08-01

**Authors:** J. Rafael Montenegro-Burke, Christopher J. Woldstad, Mingliang Fang, Aditya N. Bade, JoEllyn McMillan, Benson Edagwa, Michael D. Boska, Howard E. Gendelman, Gary Siuzdak

**Affiliations:** 1 Scripps Center for Metabolomics and Mass Spectrometry, The Scripps Research Institute, 10550 North Torrey Pines Road, La Jolla, CA 92037 USA; 2 The Department of Pharmacology and Experimental Neuroscience, University of Nebraska Medical Center, Omaha, NE 68198-5880 USA; 3 The Department of Radiology, University of Nebraska Medical Center, Omaha, NE 68198-1045 USA; 4 Department of Molecular and Computational Biology, The Scripps Research Institute, 10550 North Torrey Pines Road, La Jolla, CA 92037 USA

**Keywords:** Metabolomics, Antiretroviral therapy, Human immunodeficiency virus (HIV), Neurotoxicity, Nanomedicine, Neuroprotection

## Abstract

Antiretroviral therapy (ART) restricts human immunodeficiency virus type one (HIV-1) replication and by so doing, improves the quality and longevity of life for infected people. Nonetheless, treatment can also lead to adverse clinical outcomes such as drug resistance and systemic adverse events. Both could be affected by long-acting slow effective release ART. Indeed, maintenance of sustained plasma drug levels, for weeks or months, after a single high-level dosing, could improve regimen adherence but, at the same time, affect systemic toxicities. Of these, the most troubling are those that affect the central nervous system (CNS). To address this, dolutegravir (Tivicay, DTG), a potent and durable HIV integrase inhibitor used effectively in combination ART was tested. Rodents were administered parenteral 45-mg/kg doses. DTG-associated changes in CNS homeostasis were assessed by measuring brain metabolic activities. After antiretroviral treatment, brain subregions were dissected and screened by mass spectrometry-based metabolomics. Metabolic drug-related dysregulation of energy and oxidative stress were readily observed within the cerebellum and frontal cortex following native drug administrations. Each was associated with alterations in neural homeostasis and depleted canonical oxidation protection pools that included glutathione and ascorbic acid. Surprisingly, the oxidative stress-related metabolites were completely attenuated when DTG was administered as nanoformulations. These data demonstrate the importance of formulation design in control of DTG or perhaps other antiretroviral drug-associated CNS events.

**Electronic supplementary material:**

The online version of this article (10.1007/s12035-018-1273-8) contains supplementary material, which is available to authorized users.

## Introduction

Human immunodeficiency virus type one (HIV-1) infection is associated with a spectrum of comorbid conditions that includes the central nervous system (CNS) [1–3]. Nonetheless, many of these conditions have been either reduced or eliminated following the widespread use of antiretroviral therapy (ART). ART has enabled infected people to live nearly normal lives by reducing plasma and tissue HIV-1 RNA to levels below the limits of detection [4, 5]. Nonetheless, disease morbidities remain, albeit at lower levels, both as a consequence of viral infection and concomitant illnesses. This includes abuse of illicit drugs, nutritional deficiencies, neuropsychiatric disorders, and a variety of comorbid infectious diseases that include hepatitis and opportunistic viral and parasitic infections. Malignancies, depression, and immune dysfunctions are specifically linked to cognitive and behavioral abnormalities as are antiretroviral drugs (ARVs) themselves. The latter plays a role in direct neurotoxicity and consequent neuropsychiatric compromise [6, 7]. Among them, to date, efavirenz is known to affect neuronal function [8]. The potential effects that other ARVs may have on brain function and metabolism remain poorly understood.

Such drug-associated toxicities could become more pronounced as commonly used ARVs are transformed into long-acting slow effective release ART (LASER ART). Such achievements have been made possible by chemical drug modifications enabling lipophilic hydrophobic nanocrystals to be created that markedly prolong the drug’s apparent half-life ([9, 10]). One of the ARVs, dolutegravir (DTG), has received notable attention for its potential for transformation into a long-acting drug as well as its potential albeit rare toxicities [11]. In 2013, DTG was approved by the U.S. Food and Drug Administration [12] and soon afterwards broadly incorporated in combination ART regimens based on its potency and limited viral resistance patterns [13, 14]. While the drug is generally well tolerated, reports have emerged that in some aged patients and females, adverse events could signal off-target drug effects involving the central nervous system (CNS) [11]. Given DTG’s key role in HIV-1 therapeutic regimens, studying the intricate metabolic mechanism of how and why neural effects occur could improve disease outcomes. Further, as DTG use increases among older patients, the knowledge of who could be at risk and any means to prevent it appears timely. The realization of LASER ART could, in part, facilitate any untoward drug effects as the circulating drug half-life broadens further. Alternatively, nanoformulated drug improvements in ARV uptake, retention, and biodistribution into tissues and organs where free drug may not easily reach could attenuate adverse events by further reducing the impact of virus on disease events and in control of untoward macrophage inflammatory responses.

Indeed, while LASER ART can significantly improve antiretroviral clinical potency [15–19], it requires high doses to maintain the required four times the effective dose 90 in plasma to elicit sustained viral restriction ([9, 10]). With this in mind, we investigated the metabolic effects of free and nanoformulated DTG in five different anatomical brain regions using untargeted and unbiased metabolomic detections. DTG was administered intramuscularly at 45-mg/kg doses over a 7-day time course. This enables up to 100 times the oral therapeutic plasma concentration. Reported steady-state plasma concentration obtained from HIV-infected adults after ten doses of monotherapy with 50 mg (once daily) DTG tablets is 3.34 μg/mL (*C*_max_) [20, 21], whereas in the current study, plasma concentration of injectable DTG for a single free DTG injection was up to 11.29 μg/mL. The frontal cortex (FC), ventral cortex (VC), dorsal cortex (DC), hippocampus (H), and cerebellum (CR) were dissected and then analyzed using liquid chromatography-mass spectrometry (LC-MS)-based global metabolomics. The results showed that “high parenteral doses” of free DTG induced disordered brain metabolism as defined by increases in specific energy-related metabolites and their pathways all linked to oxidative cellular processes. These were seen predominantly in the cerebellum and cortex and were attenuated to basal levels by the use of DTG delivered as a nanoformulation. While higher dosages of DTG could affect neurotoxicities, any concern in such administration as a long-acting antiretroviral was abrogated by an established nanoformulated drug delivery system.

## Methods

### Preparation and Characterization of Nanoformulated DTG

For preparation of P407-DTG, 0.5% (*w*/*v*) P407 was mixed with 1% drug. The suspensions were homogenized at 20,000 psi using an Avestin Emulsiflex C3 homogenizer (Avestin Inc., Ottawa, ON, Canada) until the desired particle size (300–400 nm) was reached. Free polymers and nonencapsulated drug particles were removed by centrifugation; the nanoparticles were resuspended in 0.2% P407. Drug loading was determined using reversed-phase high-performance liquid chromatography (HPLC) and ultra-performance liquid chromatography tandem mass spectrometry (UPLC-MS/MS) as described. Particle size, polydispersity, and zeta potential for the nanoformulations were determined by dynamic light scattering using a Malvern Zetasizer Nano-ZS instrument (Malvern Instruments Inc., Westborough, MA, USA).

### DTG Injection and Brain Tissue Collection

Both nanoformulated and free DTG with their corresponding controls (vehicle only) were introduced by intramuscular injections with a loading dose of 45 mg/kg. Animals treated with nanoformulations were treated once while the free DTG group was treated every other day over a 1-week span (i.e., 4 total injections over 7 days) to maintain drug levels in the animals. Free DTG was dissolved in a buffer of (*v*/*v*) 43% ethanol, 5% cremophor, 20% propylene glycol (propane-1,2-diol), and 32% PBS. In the global metabolomics analysis, the same buffer and nanoformulation without DTG were used as controls. Mice were anesthetized with 1–2% isoflurane in oxygen and then aligned in a water-jacketed holder for microwave irradiation with a Muromachi Microwave Fixation System (10-kW model, Muromachi Kikai Co., Ltd., Chuo-ku, Tokyo, Japan). Irradiation time was 800 ms at 4.9 kW [22]. Single voxel localized spectra were acquired post-mortem at the midbrain to ensure metabolite level stabilization using point-resolved spectroscopy. Spectra were acquired with a repetition time of 4 s and echo time of 50 ms, 128 averages, using birdcage coil transmit, and received on a 7-Tesla/16-cm Bruker Pharmascan (Karlsure, Germany) MRI/MRS system. Single-scan, localized, unsuppressed water signals were acquired as a reference for metabolite normalization. Brains with abnormal N-acetylaspartate (NAA) or lactate concentrations were eliminated from further analysis. Five animals were selected for further brain dissection and analysis. All specimens were from the same genetic strain (Balb/cJ), males and similar ages (7 weeks). After spectroscopic validation of microwave irradiation euthanasia, brains were isolated and initially split into hemispheres, with both hemispheres dissected into subregions. Subregional dissection followed anatomical boundaries to separate the frontal cortex, ventral cortex, dorsal cortex, hippocampus, and cerebellum. Following dissection, all tissues were flash frozen in dry ice and stored at − 80 °C.

### Metabolome Extraction

Brain tissue subregions were extracted as previously described [23]. Briefly, 0.6 mL of cold methanol:H_2_O (4:1, *v*/*v*) were added per 10 mg tissue (solvent volume was adjusted accordingly to tissue weight). Homogenization was performed with glass beads in a homogenizer and sonicated in an ice bath for 10 min. The mixtures were then transferred to 1.5-mL Eppendorf vials and rinsed with additional 200 μL extraction solvent. To precipitate proteins, the samples were incubated for 1 h at − 20 °C, followed by a 15-min centrifugation at 13,000 rpm at 4 °C. The resulting supernatant was evaporated to dryness in a vacuum concentrator. The dry extracts were then reconstituted in acetonitrile:H_2_O (1:1, *v*/*v*), normalized by tissue weight, sonicated for 10 min, and centrifuged for 15 min at 13000 rpm and 4 °C to remove insoluble debris. The supernatants were transferred to HPLC vials and stored at − 80 °C prior to LC-MS analysis.

### HILIC-MS and Data Analysis

The extracts were analyzed on a 6550 iFunnel QTOF mass spectrometer coupled with a 1290 UPLC system (Agilent Technologies, Santa Clara, CA). For global metabolomics, a Luna Aminopropyl, 3 μm, 150 mm × 2.0 mm I.D. HILIC column (Phenomenex, Torrance, CA) was used. The mobile phase was composed of A = 20 mM ammonium acetate and 40 mM ammonium hydroxide in 5% ACN and B = 95% acetonitrile. A linear gradient from 100% B (0–2 min) to 100% A (17–33 min) was applied with a 15-min re-equilibration time. The flow rate and injection volume were 250 μL/min and 5 μL, respectively. ESI source conditions were set as follows: dry gas temperature, 200 °C; flow, 11 L/min, fragmentor, 380 V; sheath gas temperature, 300 °C; flow, 9 L/min; nozzle voltage, 500 V; and capillary voltage, − 500 V in ESI-negative mode. The instrument was set to acquire data over the *m/z* range 50–1000, with the MS acquisition rate of 1 spectra/s. The sample sequence was randomized to avoid systematic decreases in signals over sample sets. For the MS/MS of selected precursors, the default isolation width was set as narrow (∼ 1.3 *m/z*), with a MS acquisition rate at 2 spectra/s and MS/MS acquisition at 2 spectra/s to acquire over the *m/z* range 50–1000 and 25–1000; respectively. MS/MS data were acquired at the collision energy of 20 V.

LC-MS data were converted to mzXML files using Masshunter Acquisition Software (Agilent Masshunter 6.0B). The mzXML files were uploaded to XCMS Online web platform for data processing (https://xcmsonline.scripps.edu) including peak detection, retention time correction, profile alignment, and isotope annotation [24]. Data were processed using both pairwise and multigroup comparisons, and the parameter settings were as follows: centWave for feature detection (Δ *m/z* = 15 ppm, minimum peak width = 10 s, and maximum peak width = 60 s); obiwarp settings for retention time correction (profStep = 0.5); and parameters for chromatogram alignment, including mzwid = 0.015, minfrac = 0.5, and bw = 5. The relative quantification of metabolite features was based on extracted ion chromatogram areas. Paired parametric *t* test and one-way ANOVA (post hoc Tukey test) were used to test the variation pattern of metabolite features between and across cell samples. The result outputs, including EICs, pairwise/multigroup cloud plot, multidimensional scaling plots, and principle components, were exported directly from XCMS Online. Generally, the numbers of total pairwise comparison features and significantly altered features (statistically defined as *p* value < 0.01, including both upregulated and downregulated features) were reported in this study.

### MDM and Neuronal Culture Assays

Human monocyte-derived macrophages (MDM) were isolated and then differentiated as described [25]. MDM were cultured in Dulbeccos Modified Eagle medium (Invitrogen) supplemented with 2 mM l-glutamine, 100 μg mL^−1^ streptomycin, 100 U mL^−1^ penicillin, and 2% fetal calf serum. Murine neurons were isolated from E16-E17 (embryonic) mouse whole brain tissues. Briefly, embryonic day 16 mice were harvested by cesarean section from anesthetized pregnant dams (C57BL/6 strain). The animal protocol was approved by the Animal Care and Use Committee of University of Nebraska Medical Center. Whole brain tissues were isolated and dissociated by 10% (*v*/*v*) trypsin (Life Technologies, Bethesda, MD) digestion and trituration with a fire-polished Pasteur pipette. Cell culture dishes were coated with 33 μg/mL poly-d-lysine. The cells were plated in neurobasal medium supplemented with B27, 300 μM glutamine, 25 μM mercaptoethanol, and streptomycin/amphotericin B (Life Technologies, Waltham, MA). Three days after plating, 50% of the medium was changed and subsequently the medium was changed every 6 days. Cells were maintained in the culture for 8–10 days for complete differentiation before any treatment.

### Cell-Based DTG Measurements

MDM and neuron cells with different treatments were rinsed with phosphate-buffered saline (PBS) and extracted with acetonitrile:H_2_O:methanol (2:1:2, *v*/*v*/*v*) using freeze-thaw method as described elsewhere [26]. The samples were centrifuged at 16,000*g* at 4 °C for 15 min and the supernatant was directly injected into the triple-quad 6495 (Agilent Technologies, Santa Clara, CA) operated in multiple reaction monitoring mode (MRM), where the collision energies and product ions (MS2 or quantifier and qualifier ion transitions) were pre-optimized (quantifier ion 420 to 295 and qualifier ion 420–277). Cycle time was 150 ms for each transition. ESI source conditions were set as following: gas temperature 250 °C, gas flow 14 L/min, nebulizer 20 psi, sheath gas 250 °C, sheath gas flow 11 L/min, capillary voltage 3000 V, nozzle voltage 1500 V, and EMV 1000 V in ESI-positive mode. The analyses were performed on a Waters UPLC BEH Amide column (50 × 1 mm, 1.7 μm) (Waters Corporation, Milford. MA). The mobile phase was composed of A = water with 20 mM ammonium formate and 0.1% formic acid and B = acetonitrile with 0.1% formic acid. A linear gradient from 5% B (0–0.5 min) to 30% B (0.5–5 min, maintaining for 2 min) was applied. Then, the gradient was set to the initial 5% B within the next 1 min. A 2-min re-equilibration time was applied to the column for re-equilibration. The flow rate was 200 μL/min, and the sample injection volume was 2 μL.

### Reactive Oxygen Species (ROS) Measurements

ROS were measured in neuron and MDM cells with the probe DCFDA (dichlorofluorescin diacetate, abcam, Cambridge, MA). Cells were seeded on black, clear-bottomed 96-well plates. After cells were pre-cultured with different treatments, the media were removed and the cells were washed with 1× buffer (supplied with the kit). Cells were incubated for 45 min in 1× buffer containing 25 μM DCFDA at 37 °C. DCF production was measured by fluorescence spectroscopy with excitation wavelength at 485 nm and emission wavelength at 535 nm. In this study, the cells were treated with increasing concentrations of free DTG, nano-DTG, and various vehicle, positive, and negative controls to compare ROS generation and lactate dehydrogenase (LDH)-based cytotoxicity generated by such treatments. The highest DTG levels measured in the mouse brain is in the micromolar range so the highest dosing concentration used for ROS experiment was set to 500 μM in the dosing medium, which did not show significant cytotoxicity. TBHP (tert-butyl hydroperoxide; 10 μM) and PBS were used as the positive and negative controls, respectively. The vehicle control for nanoformulated DTG was composed of poloxamer micelles without the drug. The vehicle control for free DTG was composed of 43% ethanol, 5% cremophor, 20% propylene glycol (propane-1,2-diol), and 32% PBS (*v*/*v*). To further validate the protective effect of the P407 nanoformulation in mediating the oxidative stress, we conducted additional experiments to measure ROS formation in neurons and MDM cells treated with free and nanoformulated DTG with a series of dilutions from 10 to 100 μM of drug and then challenged with the positive control 10 μM TBHP to induce further oxidative stress. Specifically, cells (both MDM and neurons) were treated for 2 h with various vehicle or drug concentrations. Then, cells were washed and new media was added, and after 24-h culture, cells were challenged with TBHP for 2 h, followed by fluorescence-based ROS measurements.

## Results

### Brain Region-Specific Metabolomics After Free and Nanoformulated DTG Injections

**Fig. 1:**
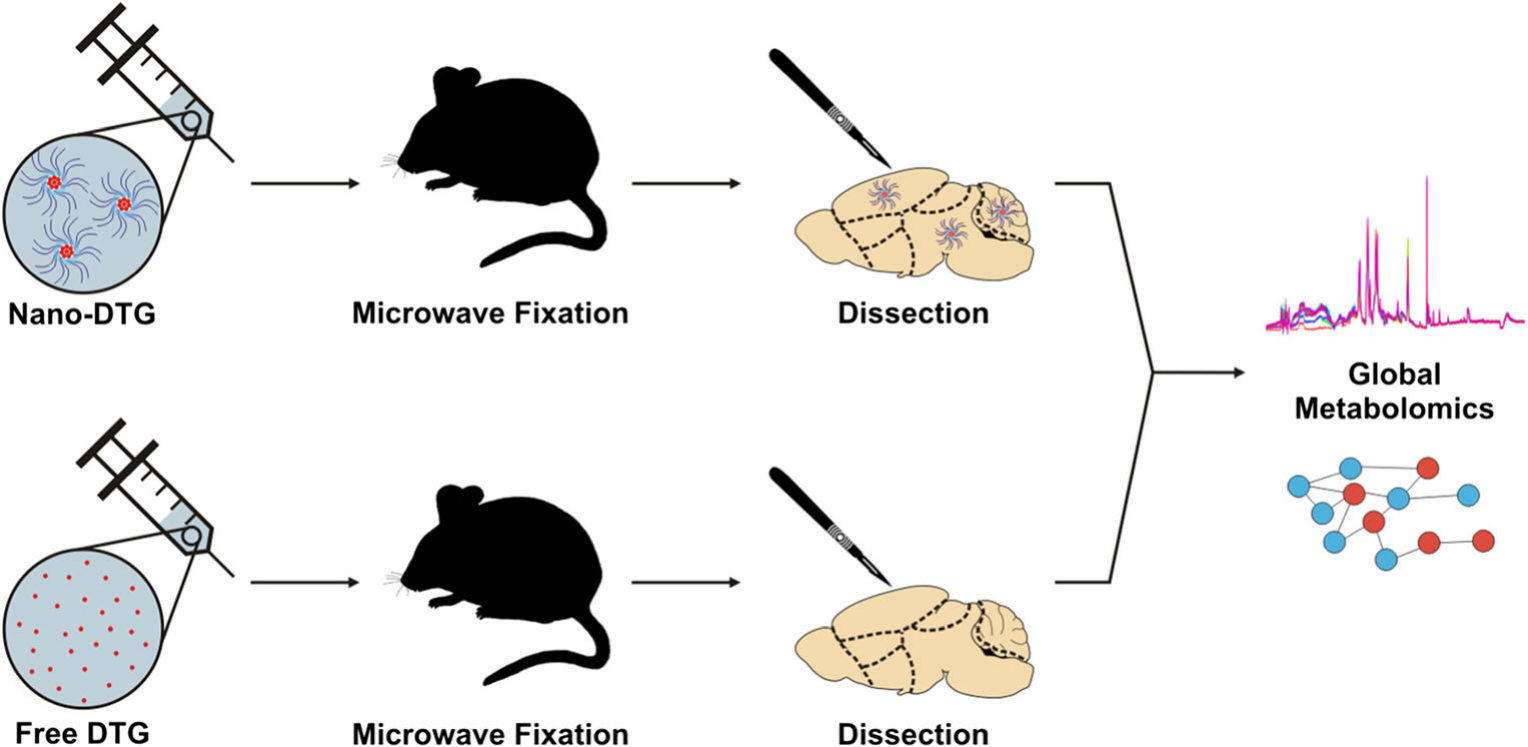
Workflow of mouse brain global metabolomics study with free and nanoformulated DTG with drug administered every other day for three total injections or by a single bolus of 45 mg/kg, respectively. All mice were sacrificed after 1 week following treatment. After heat fixation by microwave irradiation euthanasia, brain hemispheres were dissected into subregions including the FC, the VC, the DC, the H, and the CR. Brain subregions were extracted for untargeted LC-MS metabolic profiling (mice specimens = 5, total 50 sub-samples)

**Fig. 2:**
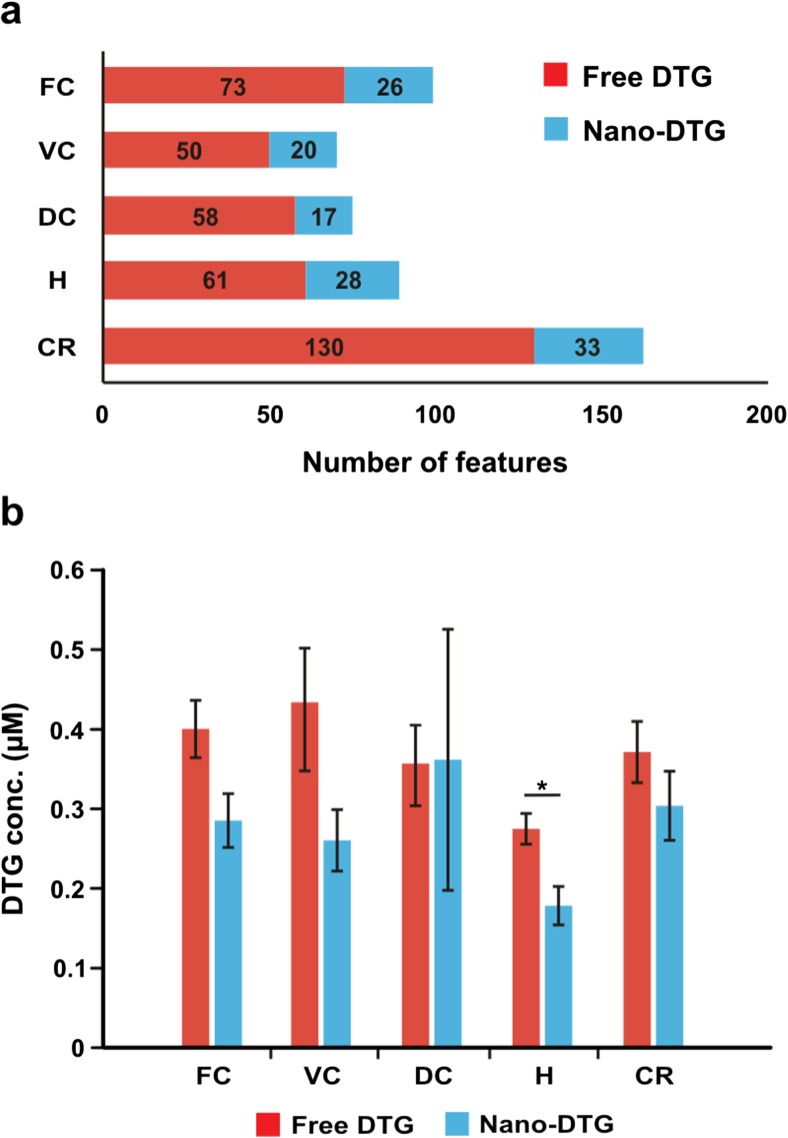
**a** Total number of dysregulated features among the FC, VC, DC, H, and CR. All the features were manually filtered. **b** DTG concentration in brain regions for free and nanoformulated DTG (nano-DTG) (error represents SEM and “*” represents *p* value < 0.05). For each experiment, a minimum of four experimental replicates were used

Following free and nanoformulated DTG mouse injections, global metabolomics were performed on five dissected brain subregions including the FC, VC, DC, H, and CR (Fig. 1). The effects of free DTG on the brain metabolome were more pronounced within the FC and CR (Fig. 2a). In free DTG-treated mice, the FC and CR showed the most pronounced metabolite dysregulations with 130 and 73 features in comparison to 50, 58, and 61 for the VC, DC, and H. Each of the numbers listed are posted in comparison to controls. The dysregulated metabolites observed in the CR overlapped, in part, with those observed in the other four brain subregions. In contrast to the metabolite effects seen by free DTG, nanoformulated DTG-treated animals showed few dysregulated features. Interestingly, the number of dysregulated features had a narrower range from 33 to 17, which on average represents a third of the number in the free DTG treatment group. Furthermore, equivalent DTG drug concentrations were measured in all five brain regions in mice treated with either free or nanoformulated DTG, demonstrating the advantages of increased macrophage uptake and retention and apparent plasma drug half-life of the nanoparticle delivered DTG compared to free drug as a delivery system. Statistical significant differences in DTG concentrations were only observed in the H, where lower drug concentrations were measured in the nanoformulated drug-treated mice. However, the fold change (0.63) was valued as small and unlikely to affect the observed metabolite response differences (Fig. 2b). Indeed, DTG nanoparticles are rapidly endocytosed by macrophages and release drug in a controlled manner in tissue target locations, which more likely reflects the differences seen.

**Fig. 3:**
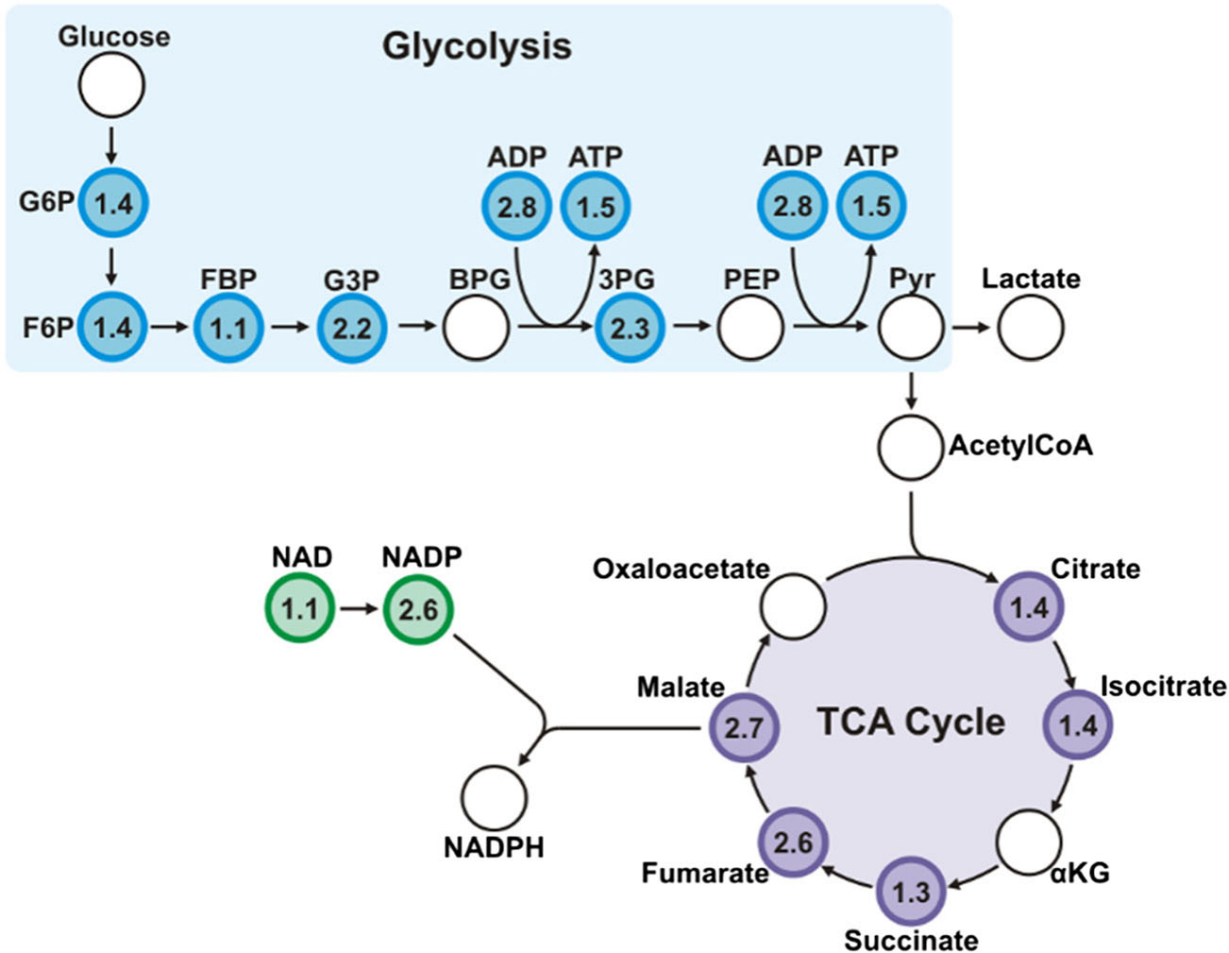
Glycolysis/TCA pathways and redox partners with identified metabolites as well as their fold change between free DTG and the corresponding control. Those values in circles had a *p* value < 0.05. All the metabolites were identified using METLIN MS/MS fragment match and confirmed with their pure standards

Among the many dysregulated metabolites seen in brain subregions in the free DTG-treated group, the most affected were identified as energy-related pathways, including glycolysis and the tricarboxylic acid (TCA) cycle (Fig. 3). Also, the dysregulation of nicotinamide adenine dinucleotide (NAD) and nicotinamide adenine dinucleotide phosphate (NADP), known cofactors in redox reactions, proved highly relevant due to linkages to oxidative stress related to xenobiotics and environmental pollutants affecting biological systems. The dysregulation of these was not observed between controls and DTG nanoparticles, which suggests negligible drug brain metabolic disruption when DTG was encased in nanoformulations.

### DTG and Oxidative Stress

**Fig. 4:**
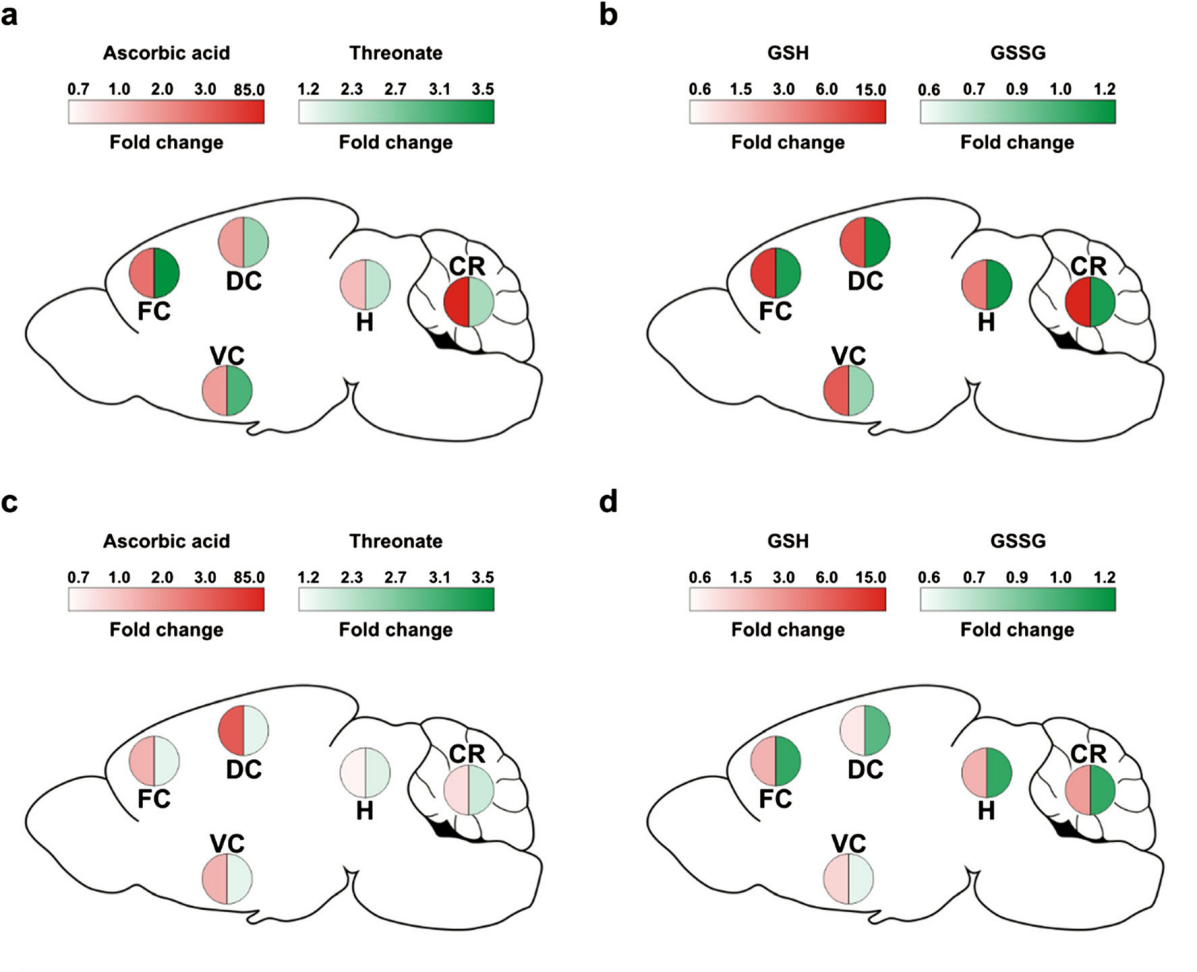
Fold change of metabolites indicative of oxidative stress in five different brain regions for both free DTG and nanoformulated DTG treatments with their corresponding controls. Ascorbic acid is oxidized to threonate and GSH is oxidized to GSSG. The color scheme was selected to show oxidation with darker shades of red for ascorbic acid and GSH and green for threonate and GSSG. **a** Ascorbic acid and threonate fold changes for free DTG treatment. **b** GSH and GSSG fold changes for free DTG treatment. **c** Ascorbic acid and threonate fold changes for nanoformulated DTG treatment. **d** GSH and GSSG fold changes for nanoformulated DTG treatment (*n* = 10 samples from 5 mice). All fold changes in **a** and **b** except for H in **a** have *p* values < 0.05. None were found to be statistically significant in **c** and **d**. The experiments listed represent a minimum of four replicates for each group

In addition to energy-related pathways, the most significant change in DTG-induced brain metabolism was in ascorbic acid and glutathione degradation. These changes are depicted in Fig. 4 for both free (a, b) and nanoformulated (c, d) DTG in each brain subregion. These values represent the ratio of the amount found in the controls divided by the amount found in the treated mice for ascorbic acid and GSH, as they are depleted with oxidative stress. Conversely, the values for threonate and GSSG represent the ratio of the amount found in the treated mice divided by the amount found in their corresponding controls. In these assays, higher ratios reflect greater degrees of oxidative stress seen as visual darker shades with oxidation, red for depletion of protecting metabolites (ascorbic acid and GSH) and green for the increase in oxidation products (threonate and GSSG).

The most substantial changes in ascorbic acid levels were observed in the FC and CR in animals treated with free DTG. Similarly, increases in threonate further support the presence of oxidative stress (Fig. 4a). Analogous to ascorbic acid and threonate metabolic dysregulation in free DTG treatment groups, GSH and GSSG showed similar effects with the exception of increases in oxidative stress seen across each of the five brain regions (Fig. 4b). All changes found in free DTG groups are statistically significant (*p* value < 0.05) with the exception of ascorbic acid levels observed in the H (Sup. Fig. 2a–d). In contrast to free DTG, nanoformulated DTG-treated animals showed less ROS brain-associated metabolic dysregulation. In Fig. 4c, d, more limited changes were seen without statistical significance compared to controls. It should be noted that similar brain drug levels between free and nanoformulated drugs were seen across both formulations and as such cannot explain the experimental differences (Fig. 2b). To investigate the metabolic source of the oxidative stress and the association with nanoformulation, a number of mechanistic laboratory experiments were performed for cross validation and extension.

### Nanoparticle DTG Encasement Abrogates Brain Oxidative Stress

**Fig. 5:**
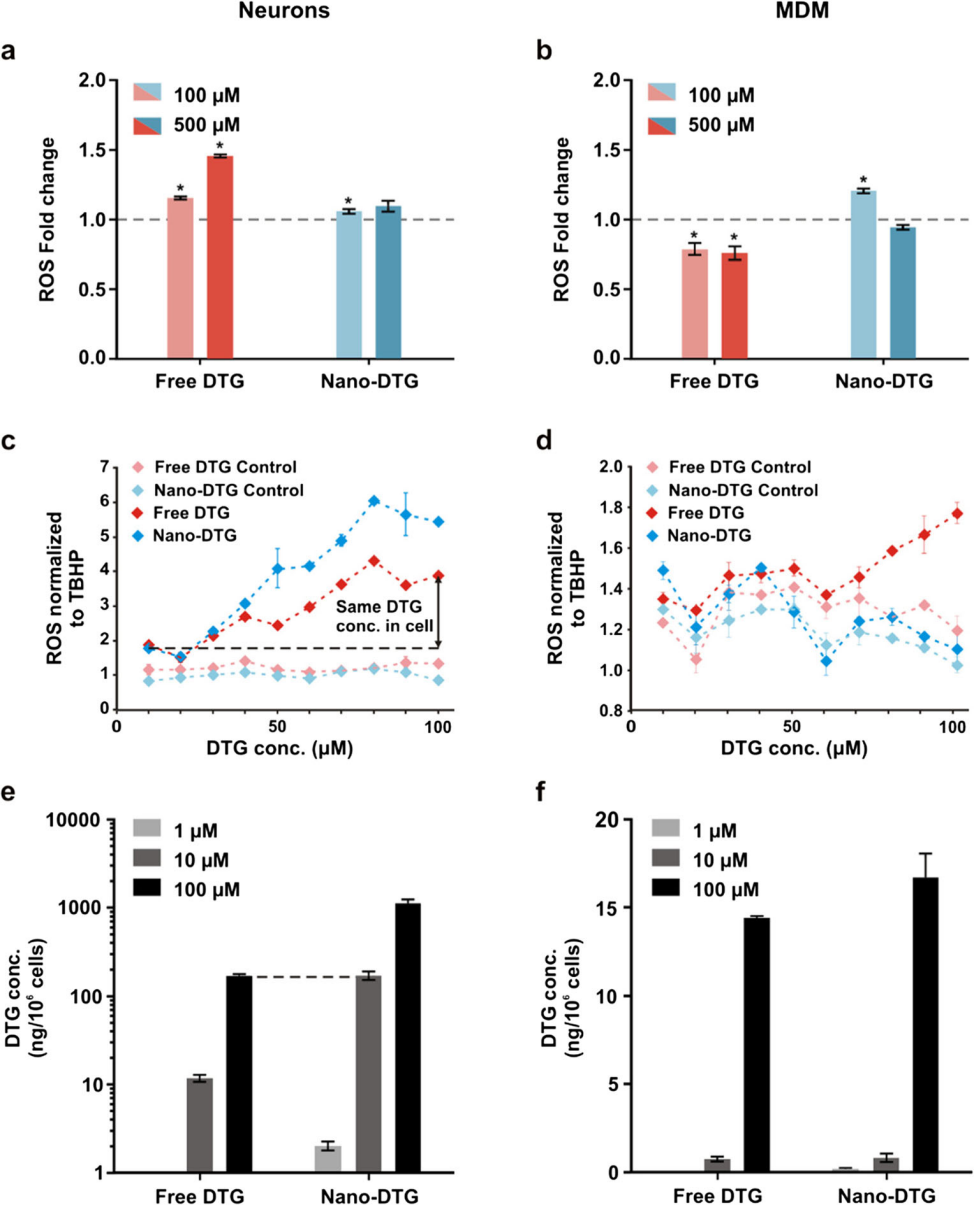
ROS formation and drug uptake of free and nanoformulated DTG in neurons and MDM cultures. **a**, **b** ROS fold change of neurons and MDM relative to their respective controls, respectively (error represent SEM and “*” represents *p* value < 0.01). **c**, **d** ROS levels after TBHP challenge in the neurons and MDM treated with free DTG, nanoformulated DTG, and their respective vehicle controls with a series of dilution from 10 to 100 μM. The data was normalized with TBHP-positive control (10 μM). **e**, **f** DTG concentration in neurons and MDM respectively treated with free DTG and nanoformulated DTG. The DTG concentration in neurons treated with 100 μM free DTG equals the DTG concentration treated with 10 μM nanoformulated DTG (dotted line, 168 ng/10^6^ cells). This comparison by cellular uptake is indicated by the dotted line in **c**

In attempts to elucidate any neuroprotective mechanism underlying the DTG nanoformulations, we employed mouse embryonic neuron cells and human MDM for testing. Each was treated with free and nanoformulated DTG with the addition of their respective vehicle controls. ROS formation was measured orthogonally in neurons and MDM using fluorescence-based commercial kits (DCFDA, Fig. 5a, b, respectively) and administered at 100 and 500 μM. Values are reported as fold changes compared to respective vehicle controls. Neurons treated with free but not nanoformulated DTG showed increases in ROS of up to 1.5 times of control. In contrast for MDM, free and nanoformulated DTG induced more limited ROS effects altering metabolite concentrations 0.8 and 1.2 times, respectively, when given at 100 μM.

The abilities of nanoparticles to modestly increase ROS suggested that P407 by itself is not an antioxidant but that the protective mechanism could be more physically linked. ROS levels in free DTG cell cultures were higher than those in the vehicle controls as aligned with the notion that DTG can itself induce oxidative stress. More interestingly, comparisons between free and nanoformulated DTG supported the hypothesis that nanoformulation can reduce oxidative stress and protect neurons (Sup. Fig. 3a–d). In attempts to sort out these differences and to better elucidate the effects of free and nanoformulated DTG and relevant drug delivery vehicles on ROS induction, we performed dose-response ROS induction following oxidative stress induced by TBHP. In these experiments, neuron and MDM cells were treated with both free and nanoformulated DTG (concentration between 10 and 100 μM) followed by the addition of 10 μM TBHP as an oxidative stress inducer. As shown in Fig. 5c, a clear ROS response was seen in neurons treated with either free or nanoformulated DTG, while no effect was seen for vehicle controls. Most importantly, for MDM (Fig. 5d), free DTG challenged with TBHP induced ROS formation in a dose-response at higher concentrations (60 to 100 μM), reflective of what was observed previously in neurons. In contrast, addition of nanoformulated DTG to the cultures attenuated the TBHP response signaling control of ROS or the ability to buffer such effects by the nanoformulations. These latter data provide a mechanism for how the nanoformulation could control ROS production in specific brain regions. Finally, we measured cellular uptake of free and nanoformulated DTG at 1, 10, and 100 μM (Fig. 5e, f) in attempts to better uncover mechanisms. As shown in Fig. 5e, the concentrations of nanoformulated DTG in neurons were almost one order of magnitude higher than those in the free DTG drug. However, for MDM, the concentration was similar between two forms of DTG treatment (Fig. 5f), which could be explained by different drug uptake mechanisms, biotransformations, and cell metabolic capacity between both cell types. However, considering the fact that DTG concentrations of nano-DTG-treated cells are one order of magnitude higher than those of free DTG-treated cells at the same dosing concentration (Fig. 5a, b), the nanoformulation shows its protective effect in modulating ROS level (e.g., fold change of 4 at 100 μM for free DTG and fold change of 1.2 at 10 μM for nano-DTG). As for MDM cells (Fig. 5f), the free DTG drug challenged with TBHP induced ROS formation with a clear concentration-response at higher concentrations (60 to 100 μM), similar to neurons. This explains, in part, the unexpected increase in ROS production during initial TBHP challenge in neurons (Fig. 5c).

## Discussion

The possible link between ART and neurotoxicities has only been postulated. Here, we investigated for the first time the effect of DTG on global metabolome during administration of free and nanoformulated drugs. The results showed that while free DTG delivered at high doses can affect the brain metabolism for oxidative stress, the effects can be curtailed by how the drug is formulated. Altogether, our results affirm that if and when DTG is used in part of a LASER ART regimen, it can be administered safely. It is acknowledged and despite restricted viral growth by cART, up to 60% of HIV-1-infected patients show HAND-related cognitive and behavioral abnormalities induced by comorbid infectious, cancerous, metabolic, and neuropsychiatric events [27]. ARVs themselves are potential contributors and most can elicit varying degrees of neurotoxic reactions. Each of these “purported” adverse effects is independent of drug class that includes nucleoside reverse transcriptase inhibitors (NRTIs, e.g., abacavir and lamivudine), non-NRTIs (e.g., efavirenz), entry inhibitors (e.g., maraviroc), inhibitors of the viral protease (e.g., atazanavir), and integrase inhibitors (INSTI, e.g., DTG). Notwithstanding, each of the drugs has revolutionized patient care and has improved longevity and disease morbidities considerably, and to the degree, HIV/AIDS is now a treatable chronic, managed disease. DTG, in particular, is used commonly in both first- and second-line therapies that have been projected by 2019 to account for 37% of the adult first-line NNRTI/INSTI market, representing 3.8 million patients [28]. Compared to NRTIs, DTG is durable and can cross the blood-brain barrier (BBB) allowing it to easily achieve therapeutic brain concentrations [29]. However, side effects of DTG are reported and include insomnia and headache as well as more significant allergic reactions and abnormal liver function in dual hepatitis virus-infected people [12]. Low concentrations of DTG can trigger suicidal death in erythrocytes characterized by cell shrinkage and membrane scrambling with phosphatidylserine translocation to the erythrocyte surface [30]. Putative neuropsychiatric effects were reported in adult patients who received DTG, raltegravir, and elvitegravir, suggesting specific toxicities linked to integrase strand transfer inhibitors [7, 11, 31–35]. Nonetheless, others have debunked such results with a sole substantiated DTG metabolic effect being a rise in serum creatinine due to tubular secretion inhibitions [36, 37].

Oxidative stress plays a principal role in the pathogenesis of neurodegenerative disorders including Alzheimer’s disease, Parkinson’s disease, amyotrophic lateral sclerosis, and HIV-associated dementia [38]. A spectrum of xenobiotics, mitochondrial dysfunction, accumulation of aberrant misfolded proteins, inflammation, and defects in protein clearance are all known to accumulate ROS. Xenobiotics can be oxidized to free radicals by cytochrome P450 (CYP) and generate ROS following the quenching of the radicals. DTG has been shown to be oxidized by CYP3A4, even though the major metabolic reaction was glucuronidation of DTG principally by metabolic reaction was glucuronidation of DTG principally (in humans) [39]. Dysregulation of glycolysis, TCA cycle, and redox cofactors could indicate a need of cells engaging in neutralizing ROS processes. Another ART drug, efavirenz, was also reported to alter mitochondrial respiration and enhance ROS generation, resulting in CNS damage [8, 40–42]. Therefore, the need for novel therapeutic strategies that attenuate neuroinflammation and protect neurons against oxidative stress is immediate for DTG. Despite the higher DTG dose used in this study over those administered to HIV infected patients the drug concentrations in brain were equivalent (Fig. 2b). Of interest no significant oxidative stress increase was observed during nanoformulated DTG treatment compared to free DTG-injected mice. Thus, it is speculated that P407 nanoformulation could play a vital role in mediating ROS formation. Several other non-ionic surfactants including polyethylene glycol (PEG) and poloxamer 188 have shown protective effects from oxidative damage in both laboratory and animal models of human disease [43–46]. For example, using a guinea pig spinal cord injury model, it has been shown that PEG could significantly decrease injury-induced ROS elevation and lipid peroxidation levels [45]. A further investigation showed that PEG is not an effective free radical scavenger nor does it have the ability to suppress xanthine oxidase, a key enzyme in generating superoxide, but can significantly accelerate and enhance the healing process to restore membrane integrity [42].

Our in vitro study suggested that surfactant poloxamer-407 could decrease the oxidative stress and cell damage. Thus, it is very likely that P407 has a beneficial effect on the drug efficacy of DTG treatment besides its role as a drug delivery system. This protective effect of non-ionic surfactant nanoformulations could have significant implications on future drug discovery, given the fact that oxidative stress is one of the most common side effects of many therapeutic drugs. Induction of oxidative stress can lead to drug failure in clinical trials or phase-out in the market in later use. Though oral ingestion of antioxidants could deem effective in counteracting such toxicity, it is not an ideal therapeutic solution due to their side effects or interactions with other medications or supplements [47–49]. Interestingly, the nanoformulated non-ionic surfactant tested in this study did not show any obvious toxicity in affecting brain metabolism. Previous studies have also found that nanoparticles can penetrate the BBB possibly via receptor-mediated endocytosis in brain capillary endothelial cells [50]. This suggests that another advantage of nanoformulations in antagonizing oxidative stress is the colocalization of nanoparticles and drug during distribution, resulting in possible in situ restoration of normal physiological conditions. Further investigation on the optimization of the material type, size, and formulation as well as its potential application for generic drug modification is needed. It is worth noting that oxidative stress and malformations in embryonic developmental including those linked to neural tube defects could occur [51–55]. Future works are certainly required to see if such relationships could occur between DTG and maternal fetal abnormalities. Indeed, following the submission of this manuscript, a potential increased risk of neural tube defects was reported in infants born to women who were taking DTG-based regimens at the time of conception [56–63]. As DTG-containing regimens would be administered to 15 million people in low- and middle-income countries, concerns for drug administration were discussed [63–67]. Thus, it is of some importance to expand research into any potential adverse effects of the medicine on fetal brain development.

In conclusion, we examined DTG brain metabolic activities using mass spectrometry-based metabolite profiling and observed significant dysregulation in energy and oxidative stress pathways; most notably, glutathione and ascorbic acid were depleted. This effect was significantly attenuated when DTG was administered as a nanoformulation. This data provides a possible pathway for the advantages of nanoformulations or other relevant therapeutic drug delivery systems to preclude systemic toxic reactions and most notably those that involve the nervous system.

## Electronic supplementary material

ESM 1 (DOCX 909 kb)

## References

[1] Ipp H, Zemlin AE, Erasmus RT, Glashoff RH (2014). Role of inflammation in HIV-1 disease progression and prognosis. Crit Rev Clin Lab Sci.

[2] Ho EL, Marra CM (2014). Central nervous system diseases due to opportunistic and coinfections. Semin Neurol.

[3] Chen MF, Gill AJ, Kolson DL (2014). Neuropathogenesis of HIV-associated neurocognitive disorders: roles for immune activation, HIV blipping and viral tropism. Curr Opin HIV AIDS.

[4] Meeker RB, Asahchop E, Power C (2014). The brain and HAART: collaborative and combative connections. Curr Opin HIV AIDS.

[5] Heaton RK, Clifford DB, Franklin DR, Woods SP, Ake C, Vaida F, Ellis RJ, Letendre SL, Marcotte TD, Atkinson JH, Rivera-Mindt M, Vigil OR, Taylor MJ, Collier AC, Marra CM, Gelman BB, McArthur JC, Morgello S, Simpson DM, McCutchan JA, Abramson I, Gamst A, Fennema-Notestine C, Jernigan TL, Wong J, Grant I, Group C (2010). HIV-associated neurocognitive disorders persist in the era of potent antiretroviral therapy: CHARTER Study. Neurology.

[6] Sanchez Ana, Kaul Marcus (2017). Neuronal Stress and Injury Caused by HIV-1, cART and Drug Abuse: Converging Contributions to HAND. Brain Sciences.

[7] Penafiel J, de Lazzari E, Padilla M, Rojas J, Gonzalez-Cordon A, Blanco JL, Blanch J, Marcos MA, Lonca M, Martinez-Rebollar M, Laguno M, Tricas A, Rodriguez A, Mallolas J, Gatell JM, Martinez E (2017). Tolerability of integrase inhibitors in a real-life setting. J Antimicrob Chemother.

[8] Tovar-y-Romo LB, Bumpus NN, Pomerantz D, Avery LB, Sacktor N, McArthur JC, Haughey NJ (2012). Dendritic spine injury induced by the 8-hydroxy metabolite of efavirenz. J Pharmacol Exp Ther.

[9] Edagwa B, McMillan J, Sillman B, Gendelman HE (2017). Long-acting slow effective release antiretroviral therapy. Expert Opin Drug Deliv.

[10] Sillman B, Bade AN, Dash PK, Bhargavan B, Kocher T, Mathews S, Su H, Kanmogne GD, Poluektova LY, Gorantla S, McMillan J, Gautam N, Alnouti Y, Edagwa B, Gendelman HE (2018). Creation of a long-acting nanoformulated dolutegravir. Nat Commun.

[11] Hoffmann C, Welz T, Sabranski M, Kolb M, Wolf E, Stellbrink HJ, Wyen C (2017). Higher rates of neuropsychiatric adverse events leading to dolutegravir discontinuation in women and older patients. HIV Med.

[12] Karmon Sharon L., Markowitz Martin (2013). Next-Generation Integrase Inhibitors. Drugs.

[13] Katlama C, Soulie C, Caby F, Denis A, Blanc C, Schneider L, Valantin MA, Tubiana R, Kirstetter M, Valdenassi E, Nguyen T, Peytavin G, Calvez V, Marcelin AG (2016). Dolutegravir as monotherapy in HIV-1-infected individuals with suppressed HIV viraemia. J Antimicrob Chemother.

[14] Brenner BG, Wainberg MA (2016). Clinical benefit of dolutegravir in HIV-1 management related to the high genetic barrier to drug resistance. Virus Res.

[15] Edagwa BJ, Zhou T, McMillan JM, Liu XM, Gendelman HE (2014). Development of HIV reservoir targeted long acting nanoformulated antiretroviral therapies. Curr Med Chem.

[16] Li T, Gendelman HE, Zhang G, Puligujja P, McMillan JM, Bronich TK, Edagwa B, Liu XM, Boska MD (2015). Magnetic resonance imaging of folic acid-coated magnetite nanoparticles reflects tissue biodistribution of long-acting antiretroviral therapy. Int J Nanomedicine.

[17] Puligujja P, Arainga M, Dash P, Palandri D, Mosley RL, Gorantla S, Poluektova L, McMillan J, Gendelman HE (2015). Pharmacodynamics of folic acid receptor targeted antiretroviral nanotherapy in HIV-1-infected humanized mice. Antivir Res.

[18] Puligujja P, McMillan J, Kendrick L, Li T, Balkundi S, Smith N, Veerubhotla RS, Edagwa BJ, Kabanov AV, Bronich T, Gendelman HE, Liu XM (2013). Macrophage folate receptor-targeted antiretroviral therapy facilitates drug entry, retention, antiretroviral activities and biodistribution for reduction of human immunodeficiency virus infections. Nanomedicine.

[19] Zhang G, Guo D, Dash PK, Arainga M, Wiederin JL, Haverland NA, Knibbe-Hollinger J, Martinez-Skinner A, Ciborowski P, Goodfellow VS, Wysocki TA, Wysocki BJ, Poluektova LY, Liu XM, McMillan JM, Gorantla S, Gelbard HA, Gendelman HE (2016). The mixed lineage kinase-3 inhibitor URMC-099 improves therapeutic outcomes for long-acting antiretroviral therapy. Nanomedicine.

[20] Min S, Sloan L, DeJesus E, Hawkins T, McCurdy L, Song I, Stroder R, Chen S, Underwood M, Fujiwara T, Piscitelli S, Lalezari J (2011). Antiviral activity, safety, and pharmacokinetics/pharmacodynamics of dolutegravir as 10-day monotherapy in HIV-1-infected adults. AIDS (London, England).

[21] Cottrell ML, Hadzic T, Kashuba AD (2013). Clinical pharmacokinetic, pharmacodynamic and drug-interaction profile of the integrase inhibitor dolutegravir. Clin Pharmacokinet.

[22] Epstein AA, Narayanasamy P, Dash PK, High R, Bathena SP, Gorantla S, Poluektova LY, Alnouti Y, Gendelman HE, Boska MD (2013). Combinatorial assessments of brain tissue metabolomics and histopathology in rodent models of human immunodeficiency virus infection. J Neuroimmune Pharmacol.

[23] Ivanisevic J, Stauch KL, Petrascheck M, Benton HP, Epstein AA, Fang M, Gorantla S, Tran M, Hoang L, Kurczy ME, Boska MD, Gendelman HE, Fox HS, Siuzdak G (2016). Metabolic drift in the aging brain. Aging.

[24] Tautenhahn R, Patti GJ, Rinehart D, Siuzdak G (2012). XCMS Online: a web-based platform to process untargeted metabolomic data. Anal Chem.

[25] Gendelman HE, Orenstein JM, Martin MA, Ferrua C, Mitra R, Phipps T, Wahl LA, Lane HC, Fauci AS, Burke DS (1988). Efficient isolation and propagation of human immunodeficiency virus on recombinant colony-stimulating factor 1-treated monocytes. J Exp Med.

[26] Ivanisevic J, Zhu ZJ, Plate L, Tautenhahn R, Chen S, O'Brien PJ, Johnson CH, Marletta MA, Patti GJ, Siuzdak G (2013). Toward ‘omic scale metabolite profiling: a dual separation-mass spectrometry approach for coverage of lipid and central carbon metabolism. Anal Chem.

[27] Saylor D, Dickens AM, Sacktor N, Haughey N, Slusher B, Pletnikov M, Mankowski JL, Brown A, Volsky DJ, McArthur JC (2016). HIV-associated neurocognitive disorder--pathogenesis and prospects for treatment. Nat Rev Neurol.

[28] Rossetti Barbara, Montagnani Francesca, De Luca Andrea (2018). Current and emerging two-drug approaches for HIV-1 therapy in ART-naïve and ART-experienced, virologically suppressed patients. Expert Opinion on Pharmacotherapy.

[29] Letendre SL, Mills AM, Tashima KT, Thomas DA, Min SS, Chen S, Song IH, Piscitelli SC, extended INGst (2014). ING116070: a study of the pharmacokinetics and antiviral activity of dolutegravir in cerebrospinal fluid in HIV-1-infected, antiretroviral therapy-naive subjects. Clin Infect Dis.

[30] Al Mamun Bhuyan A, Signoretto E, Bissinger R, Lang F (2016). Enhanced eryptosis following exposure to dolutegravir. Cell Physiol Biochem.

[31] Kheloufi F, Allemand J, Mokhtari S, Default A (2015). Psychiatric disorders after starting dolutegravir: report of four cases. AIDS (London, England).

[32] Kheloufi F, Boucherie Q, Blin O, Micallef J (2017). Neuropsychiatric events and dolutegravir in HIV patients: a worldwide issue involving a class effect. AIDS (London, England).

[33] Menard A, Montagnac C, Solas C, Meddeb L, Dhiver C, Tomei C, Ravaux I, Tissot-Dupont H, Mokhtari S, Colson P, Stein A (2017). Neuropsychiatric adverse effects on dolutegravir: an emerging concern in Europe. AIDS (London, England).

[34] Eiden C, Peyriere H, Peytavin G, Reynes J (2011). Severe insomnia related to high concentrations of raltegravir. AIDS (London, England).

[35] Harris M, Larsen G, Montaner JS (2008). Exacerbation of depression associated with starting raltegravir: a report of four cases. AIDS (London, England).

[36] Koteff J, Borland J, Chen S, Song I, Peppercorn A, Koshiba T, Cannon C, Muster H, Piscitelli SC (2013). A phase 1 study to evaluate the effect of dolutegravir on renal function via measurement of iohexol and para-aminohippurate clearance in healthy subjects. Br J Clin Pharmacol.

[37] Raffi F, Rachlis A, Stellbrink HJ, Hardy WD, Torti C, Orkin C, Bloch M, Podzamczer D, Pokrovsky V, Pulido F, Almond S, Margolis D, Brennan C, Min S (2013). Once-daily dolutegravir versus raltegravir in antiretroviral-naive adults with HIV-1 infection: 48 week results from the randomised, double-blind, non-inferiority SPRING-2 study. Lancet (London, England).

[38] Reynolds A, Laurie C, Mosley RL, Gendelman HE (2007). Oxidative stress and the pathogenesis of neurodegenerative disorders. Int Rev Neurobiol.

[39] Castellino S, Moss L, Wagner D, Borland J, Song I, Chen S, Lou Y, Min SS, Goljer I, Culp A, Piscitelli SC, Savina PM (2013). Metabolism, excretion, and mass balance of the HIV-1 integrase inhibitor dolutegravir in humans. Antimicrob Agents Chemother.

[40] Funes HA, Apostolova N, Alegre F, Blas-Garcia A, Alvarez A, Marti-Cabrera M, Esplugues JV (2014). Neuronal bioenergetics and acute mitochondrial dysfunction: a clue to understanding the central nervous system side effects of efavirenz. J Infect Dis.

[41] Funes HA, Blas-Garcia A, Esplugues JV, Apostolova N (2015). Efavirenz alters mitochondrial respiratory function in cultured neuron and glial cell lines. J Antimicrob Chemother.

[42] Luo J, Borgens R, Shi R (2002). Polyethylene glycol immediately repairs neuronal membranes and inhibits free radical production after acute spinal cord injury. J Neurochem.

[43] Hannig J, Zhang D, Canaday DJ, Beckett MA, Astumian RD, Weichselbaum RR, Lee RC (2000). Surfactant sealing of membranes permeabilized by ionizing radiation. Radiat Res.

[44] Lee RC, River LP, Pan FS, Ji L, Wollmann RL (1992). Surfactant-induced sealing of electropermeabilized skeletal muscle membranes in vivo. Proc Natl Acad Sci U S A.

[45] Luo J, Borgens R, Shi R (2004). Polyethylene glycol improves function and reduces oxidative stress in synaptosomal preparations following spinal cord injury. J Neurotrauma.

[46] Moloughney JG, Weisleder N (2012). Poloxamer 188 (p188) as a membrane resealing reagent in biomedical applications. Recent Pat Biotechnol.

[47] Fusco D, Colloca G, Lo Monaco MR, Cesari M (2007). Effects of antioxidant supplementation on the aging process. Clin Interv Aging.

[48] Nitta H, Kinoyama M, Watanabe A, Shirao K, Kihara H, Arai M (2007). Effects of nutritional supplementation with antioxidant vitamins and minerals and fish oil on antioxidant status and psychosocial stress in smokers: an open trial. Clin Exp Med.

[49] Bardia A, Tleyjeh IM, Cerhan JR, Sood AK, Limburg PJ, Erwin PJ, Montori VM (2008). Efficacy of antioxidant supplementation in reducing primary cancer incidence and mortality: systematic review and meta-analysis. Mayo Clin Proc.

[50] Wohlfart S, Gelperina S, Kreuter J (2012). Transport of drugs across the blood-brain barrier by nanoparticles. J Control Release.

[51] Ahmed AE, Jacob S, Campbell GA, Harirah HM, Perez-Polo JR, Johnson KM (2005). Fetal origin of adverse pregnancy outcome: the water disinfectant by-product chloroacetonitrile induces oxidative stress and apoptosis in mouse fetal brain. Brain Res Dev Brain Res.

[52] Han ZJ, Song G, Cui Y, Xia HF, Ma X (2011). Oxidative stress is implicated in arsenic-induced neural tube defects in chick embryos. Int J Dev Neurosci.

[53] Kotch LE, Chen SY, Sulik KK (1995). Ethanol-induced teratogenesis: free radical damage as a possible mechanism. Teratology.

[54] Tung EW, Winn LM (2011). Valproic acid increases formation of reactive oxygen species and induces apoptosis in postimplantation embryos: a role for oxidative stress in valproic acid-induced neural tube defects. Mol Pharmacol.

[55] Zhao Z, Reece EA (2005). Nicotine-induced embryonic malformations mediated by apoptosis from increasing intracellular calcium and oxidative stress. Birth Defects Res B Dev Reprod Toxicol.

[56] The U.S. President’s Emergency Plan for AIDS Relief (PEPFAR) (2018) PEPFAR statement on potential safety issue affecting women living with HIV using dolutegravir at the time of conception. https://www.pepfar.gov/press/releases/282221.htm.

[57] U.S. Food and Drug Administration (2018) FDA Drug Safety Communication: FDA to evaluate potential risk of neural tube birth defects with HIV medicine dolutegravir (Juluca, Tivicay, Triumeq). https://www.fda.gov/Drugs/DrugSafety/ucm608112.htm.

[58] CNBC (2018) Regulators flag possible birth defect link to GSK’s HIV drug. https://www.cnbc.com/2018/05/18/reuters-america-update-1-regulators-flag-possible-birth-defect-link-to-gsks-hiv-drug.html.

[59] World Health Organization (2018) Statement on DTG http://www.who.int/medicines/publications/drugalerts/Statement_on_DTG_18May_2018final.pdf?ua=1.

[60] REUTERS (2018) EU warns of possible birth defect link to GSK’s HIV drug. https://www.reuters.com/article/gsk-hiv-defects/eu-warns-of-possible-birth-defect-link-to-gsks-hiv-drug-idUSL5N1SP4AY

[61] U.S. Department of Health & Human Services (2018) Statement on potential safety signal in infants born to women taking dolutegravir from the HHS antiretroviral guideline panels. https://aidsinfo.nih.gov/news/2094/statement-on-potential-safety-signal-in-infants-born-to-women-taking-dolutegravir-from-the-hhs-antiretroviral-guideline-panels.

[62] The L (2018). Dolutegravir for HIV: a lesson in pregnancy safety research. Lancet (London, England).

[63] Dorward J, Lessells R, Drain PK, Naidoo K, de Oliveira T, Pillay Y, Abdool Karim SS, Garrett N (2018). Dolutegravir for first-line antiretroviral therapy in low-income and middle-income countries: uncertainties and opportunities for implementation and research. Lancet HIV.

[64] The Joint United Nations Programme on HIV/AIDS (UNAIDS) (2017) New high-quality antiretroviral therapy to be launched in South Africa, Kenya and over 90 low- and middle-income countries at reduced price. http://www.unaids.org/en/resources/presscentre/pressreleaseandstatementarchive/2017/september/20170921_TLD

[65] Hill A, Clayden P, Thorne C, Christie R, Zash R (2018). Safety and pharmacokinetics of dolutegravir in HIV-positive pregnant women: a systematic review. J Virus Erad.

[66] Clinton Health Access Initiative (2017) ARV market report, 2017. https://clintonhealthaccess.org/2017-arv-market-report/

[67] World Health Organization (2017) Transition to new antiretrovirals in HIV programmes: policy brief. http://www.who.int/hiv/pub/toolkits/transition-to-new-arv/en/

