# Supplementary material for: Nanoformulated Antiretroviral Therapy Attenuates Brain Metabolic Oxidative Stress
Source: Mol Neurobiol. 2018 Aug 1;56(4):2896–907. doi: 10.1007/s12035-018-1273-8 (PMC6403019; doi:10.1007/s12035-018-1273-8)
Supplement: Supplementary file 1 — (DOCX 909 kb) [file 12035_2018_1273_MOESM1_ESM.docx]

**Supplementary Material**

**Nanoformulated antiretroviral therapy attenuates brain metabolic oxidative stress**

J. Rafael Montenegro-Burke^1,†^, Christopher J. Woldstad^2,†^, Mingliang Fang^1^, Aditya N. Bade^2^, JoEllyn McMillan^2^, Benson Edagwa^2^, Michael D. Boska^3^, Howard E. Gendelman^2^*, and Gary Siuzdak^1,4^*

^1^Scripps Center for Metabolomics and Mass Spectrometry, The Scripps Research Institute, 10550 North Torrey Pines Road, La Jolla, California 92037, USA.

^2^The Department of Pharmacology and Experimental Neuroscience, University of Nebraska Medical Center, Omaha, Nebraska 68198-5880, USA.

^3^The Department of Radiology, University of Nebraska Medical Center, Omaha, Nebraska 68198-1045, USA.

^4^Department of Molecular and Computational Biology, The Scripps Research Institute, 10550 North Torrey Pines Road, La Jolla, California 92037, USA.

Corresponding authors:

Dr. Howard E. Gendelman, The Department of Pharmacology and Experimental Neuroscience, University of Nebraska Medical Center, Omaha, Nebraska 68198-5880, USA. Tel.:402-559-8920. Email:hegendel@unmc.edu

Dr. Gary Siuzdak, Department of Molecular and Computational Biology, The Scripps Research Institute, 10550 North Torrey Pines Road, La Jolla, California 92037, USA.  Tel.: 858-784-9415. Email:siuzdak@scripps.edu

^†^ These authors contributed equally

**Supplementary Figure 1.** Cloud plots illustrating metabolic differences between free Dolutegravir (Free DTG) and Poloxamer 407 nanoformulated DTG (Nano-DTG) with their controls in the cerebellum. **(a)** Cloud plot showing dysregulated features between Nano-DTG and control. **(b)** Cloud plot showing dysregulated features between free DTG and their controls. The features were filtered using *p* < 0.01, intensity > 10,000 and fold change > 1.5. Ascorbic acid and glutathione were indicated in the figure.


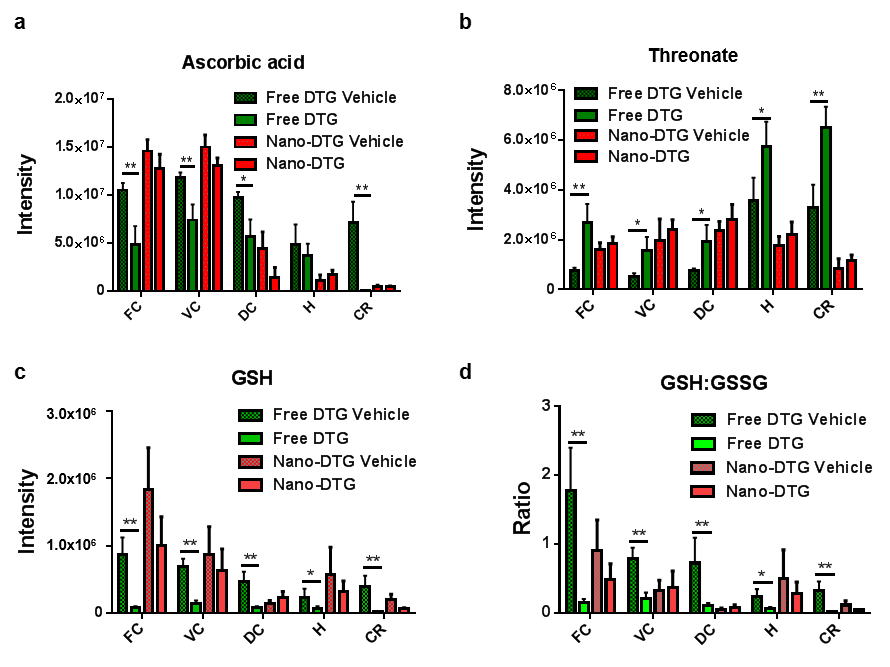


**Supplementary Figure 2**. Relative abundance of oxidative stress biomarkers. **(a)** ascorbic acid; **(b)** threonate; **(c)** glutathione (GSH); **(d)** GSH/GSSG ratio in five dissected regions, frontal cortex, ventral cortex, dorsal cortex, hippocampus and cerebellum (n=10 samples from 5 mice) from mice treated with free DTG and Nano-DTG compared with their respective controls. All data corresponds to global metabolomics analysis. Error bars represents standard error of the mean. “*” and “**” represents *p values* <0.05, and <0.01; respectively.

**Supplementary Figure 3.** Free DTG and nanoformualted DTG (Nano-DTG) reactive oxygen species (ROS) formation in murine neurons and human monocyte derived macrophage (MDM) cells 24 hours post treatment**. (a)** Neurons and **(b)** MDM treated with 10 µM TBHP (positive control), free DTG (100 and 500 µM), Nano-DTG (100 and 500 µM), and their respective vehicle controls. Error bars represent standard error of the mean (n=6).
